# The Effect of the Low Glutamate Diet on the Reduction of Psychiatric Symptoms in Veterans With Gulf War Illness: A Pilot Randomized-Controlled Trial

**DOI:** 10.3389/fpsyt.2022.926688

**Published:** 2022-06-20

**Authors:** Elizabeth T. Brandley, Anna E. Kirkland, Michael Baron, James N. Baraniuk, Kathleen F. Holton

**Affiliations:** ^1^Department of Health Studies, American University, Washington, DC, United States; ^2^Medical University of South Carolina, Charleston, SC, United States; ^3^Department of Mathematics and Statistics, American University, Washington, DC, United States; ^4^Department of Medicine, Georgetown University, Washington, DC, United States; ^5^Department of Neuroscience, American University, Washington, DC, United States; ^6^Center for Neuroscience and Behavior, American University, Washington, DC, United States

**Keywords:** anxiety, depression, PTSD, GWI, glutamate, diet, intervention

## Abstract

The objective of this pilot study was to examine the effects of the low glutamate diet on anxiety, post-traumatic stress disorder (PTSD), and depression in veterans with Gulf War Illness (GWI). The low glutamate diet removes dietary excitotoxins and increases consumption of micronutrients which are protective against glutamatergic excitotoxicity. This study was registered at ClinicalTrials.gov (NCT#03342482). Forty veterans with GWI completed psychiatric questionnaires at baseline and after 1-month following the low glutamate diet. Participants were then randomized into a double-blind, placebo-controlled crossover challenge with monosodium glutamate (MSG; a dietary excitotoxin) vs. placebo over three consecutive days per week, with assessments on day three. Data were analyzed across the full sample and with participants categorized by baseline symptom severity. Pre-post-dietary intervention change scores were analyzed with Wilcoxon signed-rank tests and paired sample *t*-tests across the full sample, and changes across symptom severity categories were analyzed using ANOVA. Crossover challenge results were analyzed with linear mixed modeling accounting for challenge material (MSG v. placebo), sequence (MSG/placebo v. placebo/MSG), period (challenge week 1 v. week 2), pre-diet baseline symptom severity category (minimal/mild, moderate, or severe), and the challenge material^*^symptom severity category interaction. A random effect of ID (sequence) was also included. All three measures showed significant improvement after 1 month on the diet, with significant differences between baseline severity categories. Individuals with severe psychological symptoms at baseline showed the most improvement after 1 month on the diet, while those with minimal/mild symptoms showed little to no change. Modeling results from the challenge period demonstrated a significant worsening of anxiety from MSG in only the most severe group, with no significant effects of MSG challenge on depression nor PTSD symptoms. These results suggest that the low glutamate diet may be an effective treatment for depression, anxiety, and PTSD, but that either (a) glutamate is only a direct cause of symptoms in anxiety, or (b) underlying nutrient intake may prevent negative psychiatric effects from glutamate exposure. Future, larger scale clinical trials are needed to confirm these findings and to further explore the potential influence of increased micronutrient intake on the improvements observed across anxiety, PTSD, and depression.

## Introduction

Approximately 700,000 US military personnel were deployed for the Gulf War (GW) (1). An estimated 25–44% of those US veterans, along with deployed veterans from other countries, developed a unique cluster of medical and psychological issues within 1 year of the war (2, 3). This multi-symptom chronic and debilitating condition has become known as Gulf War Illness (GWI) (4). It is characterized by multiple symptom domains, including persistent widespread chronic musculoskeletal pain, chronic fatigue and sleep problems, gastrointestinal issues, neurological/cognitive dysfunction, as well as respiratory problems and skin abnormalities (1, 4, 5). There are two main sets of GWI case definitions (6), one being the Kansas case definition, requiring 3 of the 6 symptom domains at the moderate-to-severe levels (4). The second, the Centers for Disease Control and Prevention (CDC) criteria, requires symptoms to be present in 2 of the 3 symptom categories of musculoskeletal pain, fatigue, and neurological issues (5).

The neurological category for both sets of criteria includes psychiatric symptoms such as anxiety, post-traumatic stress disorder (PTSD), and depression, which are shown at increased rates among GW veterans since the war ended (7). Three decades post-conflict, multiple cross-sectional and longitudinal studies found that GW veterans worldwide are still reporting increased rates of depression and anxiety disorders, including PTSD (3, 8–10). Those affected by one or more comorbid psychiatric disorders appear to be the most greatly impacted (11). Accordingly, finding successful treatment options for psychiatric conditions is of the utmost importance for this veteran population.

Gulf War Illness is hypothesized to be related to neurotoxic exposures during the GW, which may have caused central nervous system (CNS) dysregulation (12, 13). Exposures included burning oil well-fires, depleted uranium, sarin and cyclo-sarin gases, pyridostigmine bromide (PB) pills, and pesticides (14). The latter three exposures inhibit the acetylcholinesterase enzyme from breaking down acetylcholine in the synaptic cleft, thereby facilitating excessive excitation (12, 13, 15). This has downstream effects on glutamate, the most ubiquitous excitatory neurotransmitter in the body (16, 17). Overexcitation of glutamatergic neurons and prolonged activation of glutamate receptors can lead to excitotoxicity and neuronal cell death (18). Excitotoxicity can trigger neuroinflammation and oxidative stress (19–21), with each one potentially influencing the other in a self-perpetuating cycle known as the neurotoxic triad (22).

Research has demonstrated that each component of the neurotoxic triad, including glutamate excitotoxicity, oxidative stress, and neuroinflammation, have been implicated individually in the underlying pathophysiology of anxiety, PTSD, and depression (23–28). Glutamatergic neuronal projections and pathways are heavily involved in stress-related regions of the brain and can cause structural and functional changes in these regions as a result (29). When examining anxiety, PTSD, or depression in animal, clinical and post-mortem studies, structural alterations, decreased brain volume (i.e., smaller hippocampus or prefrontal cortex), changes in the expression or density of glutamate receptors, reduction in synaptic connectivity, and neuronal atrophy have all been reported (29–45). Animal studies assessing exposure to high stress or trauma have also reported astrocyte dysfunction, which can lead to extracellular glutamate levels increasing to toxic amounts (i.e., excitotoxicity) (46). Overall, these potential structural and functional abnormalities in individuals with psychiatric symptoms may disrupt glutamatergic neurotransmission and hinder mood regulation.

Glutamate is also involved in psychiatric disorders because it is involved in the release of other neurotransmitters, including monoaminergic (e.g., serotonin and dopamine) and GABAergic transmission, which are often targets of psychiatric medications (47–49). Anxiety, PTSD, and depression are related to inadequate serotonin levels (50–52) which can co-occur with glutamate dysregulation. This happens when tryptophan is pushed toward the kynurenine pathway, rather than being used for serotonin production. This process results in both reduced serotonin production as well as increased production of quinolinic acid, which functions as an agonist of the NMDA glutamate receptor (53). This redirection of tryptophan also increases glutamatergic excitation, thereby potentiating symptoms. Thus, modulation of glutamatergic neurotransmission may be important for treating symptoms of anxiety, PTSD, and depression.

Preclinical and clinical pharmacology studies have also implicated the glutamatergic system in these disorders through known and suspected drug mechanisms (23, 54–56). For example, ketamine is a non-competitive antagonist of the NMDA glutamate receptor, which has been shown to quickly reduce depressive symptoms, anxiety, and PTSD (57–61). However, the pharmacological use of ketamine appears to be somewhat limited by its side effect profile and potential for abuse (62); therefore, it may not be as applicable for long-term use. Alternative methods for modulating glutamatergic neurotransmission are needed in this veteran population.

One potential adjunct treatment for these psychiatric conditions is the low glutamate diet. Glutamate not only functions as an excitatory neurotransmitter in the body, but it is also present in the diet as an amino acid in both bound (e.g., meat) and free forms (i.e., not bound to a protein) (63). Free forms of glutamate have the ability to stimulate neurons on the tongue and are often added as flavor-enhancing food additives. Individuals with an intact blood-brain barrier (BBB) are usually protected from higher dietary intake of free glutamate due to saturation of the glutamate transporter at the BBB, thereby limiting the amount of glutamate able to access the CNS (64). However, stress, infection, traumatic brain injury (TBI), trauma, and/or neurotoxic exposures (43, 65–68), all commonly experienced by GW veterans, can cause permeability of the BBB to occur, which may allow higher quantities of dietary glutamate to cross into the brain.

It has previously been demonstrated that the low glutamate diet can successfully reduce the overall number of symptoms in veterans with GWI, including improvements in widespread pain and fatigue (69) as well as improvements in cognitive function (70). Symptom reduction has also been observed in other chronic pain populations (71, 72). However, to date, no study has evaluated the effect of the low glutamate diet on psychiatric outcomes. Thus, the objective of this research was to examine the effects the low glutamate diet on anxiety, PTSD, and depression in veterans with GWI.

## Materials and Methods

The Institutional Review Boards at both universities approved this study, in addition to the Human Research Protection Office (HRPO) of the US Army Medical Research and Materiel Command (HRPO Log NumberA-20203.a). Consent forms were electronically emailed to potential participants allowing time for review, discussion with loved ones, and ability to have all questions answered prior to signing. All participants provided written informed consent, which was obtained from each subject prior to participation. The study was registered at ClinicalTrials.gov (NCT#03342482). Details about recruitment and study design are published elsewhere (69), but will also be explained briefly below.

### Subjects

Forty-six subjects with GWI, determined by meeting both the Kansas and CDC case criteria (4, 5), were recruited from across the United States. Exclusion criteria included being a current smoker, having a substance use disorder in the past year, or a current diagnosis of epilepsy or severe asthma (due to increased risk during the challenge period) (73–75). Glutamate modulating medications were discontinued with clinician approval prior to enrollment and all other medications were kept constant (frequency/dosage) throughout the study. Six subjects dropped out of the study: one was disqualified before completing baseline assessments and two dropped out before the intervention began due to lack of computer/resources or self-efficacy. The other three participants dropped out during the intervention due to a major car accident, kidney failure, and gastric bypass surgery (see consort diagram in Supplementary Materials).

At the initial visit, subjects completed all pre-diet baseline measures, including measurement of anthropometrics and the excitotoxin food-frequency questionnaire (FFQ). Additionally, participants completed the following psychiatric measures: the Generalized Anxiety Disorder 7-item (GAD-7) instrument, the PTSD Checklist-Civilian (PLC-C), and the Center for Epidemiologic Studies Depression (CES-D) measure; all of which are considered valid and reliable measures of psychiatric symptom severity (76–78). The PCL-C was chosen for this research to better capture all events which could potentially induce PTSD symptoms, including something not related to combat such as sexual assault. The outcome measures were collected electronically to help prevent errors during data entry. Higher scores for each instrument indicated a higher likelihood of having the disorder, and cut-off scores for each were based on the accepted literature. For example, anxiety symptoms were assessed using the self-administered GAD-7 questionnaire, and were considered suggestive of having a generalized anxiety disorder if scores were ≥10 out of 21 (77). The total GAD-7 scores for each participant were categorized into 3 groups, including minimal/mild (total scores 0–9), moderate (total scores 10–14) and severe (total scores 15–21) categories (77). The PTSD symptoms, as measured by the PCL-C, were considered suggestive of having PTSD for scores ≥31 out of 85 with a range of 17–85 (79). The PCL-C total scores were divided similarly into 3 categories of minimal/mild (total scores 17–29), moderate (total scores 30–44) and severe (total scores 45–85) (80–82). Depressive symptoms, measured using the CES-D, were considered suggestive of having depression if the total score was ≥16 out of 60 (83, 84). Individual CES-D scores were also categorized into 3 groups based on total score, including minimal/mild (total scores 0–15), moderate (total scores 16–24) and severe (total scores ≥25) (84, 85).

After returning home from the pre-diet baseline assessment, all subjects completed a comprehensive 2-h online dietary training conducted by the principal investigator, detailing the low glutamate diet and describing how to read food labels and identify hidden sources of dietary excitotoxins. The diet emphasizes consumption of nutrient-dense whole foods which contain nutrients known to be protective against glutamate excitotoxicity, including foods high in omega-3 fatty acids (86), vitamin C (87–89), vitamin D (90), vitamin E (91), riboflavin (vitamin B2) (92, 93), vitamin B6 (94), zinc (95), and magnesium (96, 97). Since omega-3 fatty acids and vitamin D are chronically under consumed (98, 99) and important for reduction in glutamate excitotoxicity (86, 90), subjects were asked to consume 1 teaspoon of liquid cod liver oil daily. High-antioxidant foods were also encouraged since antioxidants are important for reducing the oxidative stress triggered by glutamatergic excitotoxicity (100, 101).

Participants also received a binder on how to follow the diet with the training information in a hard-copy format, including a list of food additives to avoid, a list of foods high in antioxidants, a detailed table of foods highest in each micronutrient according to appropriate serving sizes, a sample shopping list, and diet-approved recipes. The principal investigator was accessible to participants via phone/text message throughout the intervention period to answer any questions that arose. Participants followed the diet at home for the next month.

**Figure 1:**
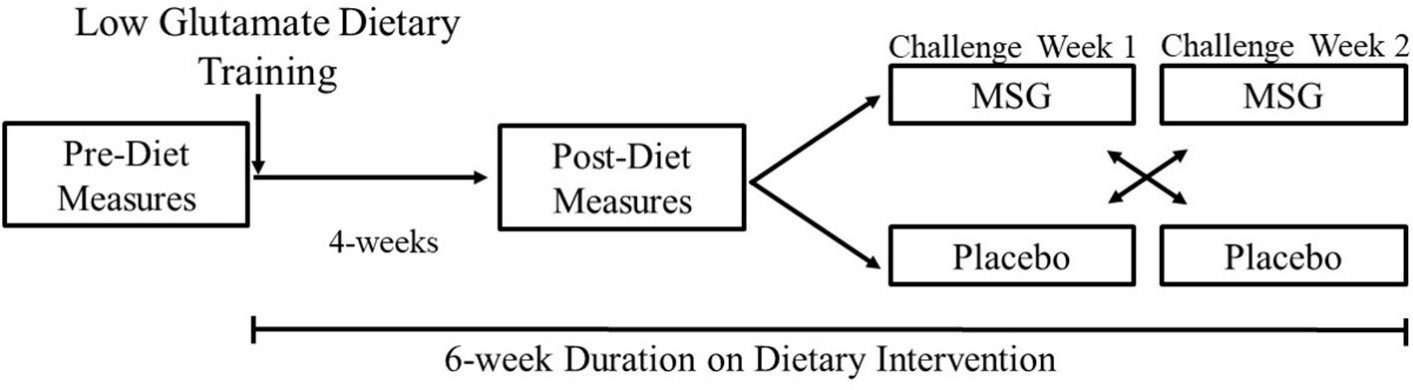
Study protocol diagram.

After 1 month on the low glutamate diet, all subjects completed a post-dietary intervention lab visit where all outcome measures were again collected. Participants were then randomized (based on computer generated randomization) into the double-blind, placebo-controlled, crossover challenge period with MSG/placebo to test for the return of symptoms upon challenge with MSG vs. placebo over the following 2 weeks. Subjects continued following the low glutamate diet during this challenge period to ensure that the only glutamate they were receiving was during the MSG challenge week. After an overnight fast, subjects received either 5 g of MSG or placebo (based on randomization) for three consecutive days in the morning and were monitored for 2 h in the lab. Participants then had a 1-week wash-out period and received the opposite contingency for three consecutive days of the following week. All measures were collected on the third day of each challenge week. The study design is illustrated in Figure 1.

Food-grade MSG manufactured by Ajinomoto was purchased commercially. All participants received 5 g of MSG regardless of weight to replicate the amount ingested by a high consumer of free glutamate. Placebo pills contained a sugar/salt mixture, with sodium levels matched to the sodium levels in the MSG. Vegetable capsules were used to avoid exposure to gelatin (as a source of excitotoxins). All research personnel were blinded except for one research assistant who created the challenge material, and this person was not involved in data collection. The blind was not broken until the data were being analyzed at the completion of the study.

### Statistical Methods

Analyses were completed using SAS^®^ 9.4. Normality of all data was assessed using the Shapiro-Wilk test. First, analyses were run to assess the effect of 1 month on the low glutamate diet across the whole sample and across pre-diet baseline symptom severity categories. Of note, while there are often 4 categories of severity for the measures evaluated in this study (i.e., minimal, mild, moderate, and severe), the minimal and mild groups were collapsed due to a small number of participants in each group. The adjustment from 4 to 3 severity groups did not impact the overall results. For the whole sample, McNemar tests were used to compare the percentage of subjects meeting the criteria for each psychiatric disorder (i.e., anxiety, PTSD, depression) at pre- and post-dietary intervention assessments. Change scores were created for all variables (i.e., post-diet minus pre-diet baseline scores) and Wilcoxon signed-rank tests or paired-sample *t*-tests were used for pre-to-post-diet comparisons for each measure, depending on passing the assumption of normality. Change scores were then compared between pre-diet baseline psychological symptom severity categories using ANOVA (dependent variable = change scores, independent variable = pre-diet baseline symptom severity categories).

Next, the double-blind, placebo-controlled crossover challenge phase of the study was assessed using linear mixed modeling. Models (without symptom severity categories) were adjusted for challenge material (MSG v. placebo), sequence (MSG/placebo v. placebo/MSG), and period (challenge week 1 v. week 2). Pre-diet baseline symptom severity category (minimal/mild, moderate, or severe) and challenge material^*^category interaction were added to test for differential response to challenge between groups. If there was a significant interaction, least squares (LS) mean differences were computed and compared using *t*-tests for the interaction term and main effects forming the term (i.e., challenge material and symptom severity category). A random effect of ID (sequence) was also included for all models. Model building was conducted for ANOVA (using ANCOVA) and linear mixed models to see if certain covariates were needed (i.e., age, sex, FFQ); these covariates did not impact the models and therefore were not included in the following results. Multiple comparisons were controlled using a more conservative Bonferroni corrected alpha level of 0.016 per test, ensuring a family-wise error rate of 0.05.

## Results

Of the forty subjects recruited for the study, 28% of the sample were women and 8% were African American (69). This sample represented multiple branches of the military including Air Force, Army, Marine Corps, and Navy (69). The average age of participants was 54 (SD = 6) years, the majority (90%) were either overweight or obese (BMI ≥25), and almost 40% of the sample were retired/disabled due to their illness (69). Overall dietary compliance, based on the excitotoxin FFQ, was excellent throughout the 6-week period (including 4-week intervention period and the 2-week challenge period) (69). No side effects were reported from the 1-month dietary intervention period. One participant failed to complete all the psychiatric questionnaires; thus, the analyses were completed on 39 subjects.

### Low Glutamate Diet (Pre-diet Baseline to Post-dietary Intervention)

At baseline, 41% of participants met the GAD-7 proposed cut-off (≥10 out of 21) for having a general anxiety disorder, 85% met the PCL-C suggested criteria (≥31 out of 85) for PTSD, and 77% of the sample met the suggested CES-D cut-off (≥16 out of 60) for depression. After 1 month on the low glutamate diet, the percentage of individuals meeting the proposed cut-off for each measure decreased to 26% for anxiety (−15% change; *p* = 0.07), 67% for PTSD (−18%, *p* = 0.02), and 54% for depression (−23% change; *p* = 0.05). There was also a significant reduction in mean scores for anxiety, PTSD, and depression (all *p* < 0.001; Table 1) across the full sample after 1-month on the dietary intervention.

**Table 1:** Change in psychological symptoms after 1-month on the low glutamate diet.

***N*** **= 39**	**Pre-diet**	**Post-diet**	***P*** **-value**
	**Mean (SD)**		
Anxiety (GAD-7)	9.5 (7.0)	6.6 (6.5)	**<0.001^a^**
PTSD (PCL-C)	52.4 (18.4)	41.8 (17.5)	**<0.001^b^**
Depression (CES-D)	27.8 (13.5)	18.7 (11.2)	**<0.001^b^**
	***N*** **(%)**		***P*** **-value^c^**
Meets criteria for anxiety	16 (41%)	10 (26%)	0.07
Meets criteria for PTSD	33 (85%)	26 (67%)	0.02
Meets criteria for depression	30 (77%)	21 (54%)	0.05

a *Wilcoxon signed-rank test*.

b *Paired-samples t-test*.

c *McNemar test. Bold, significant at Bonferroni corrected p-value of 0.016*.

**Figure 2:**
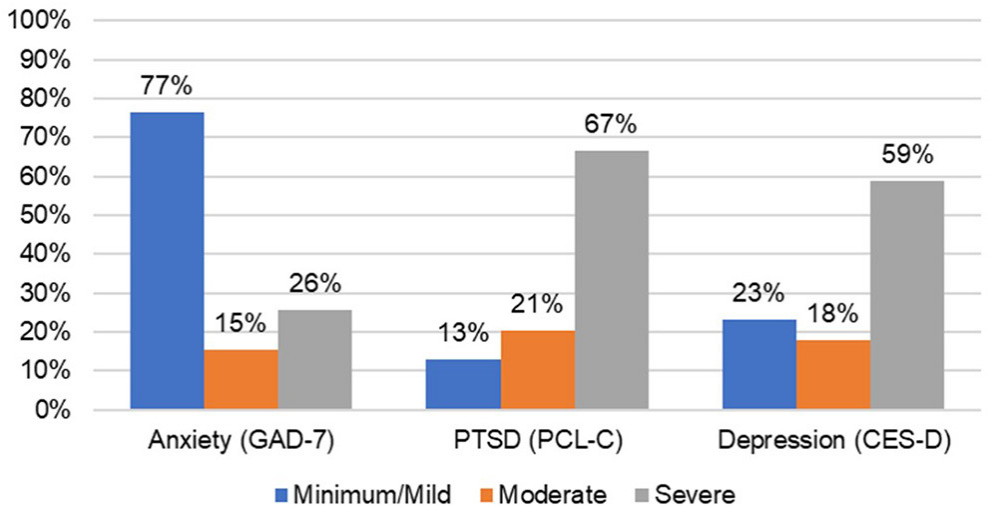
Percent of participants in symptom severity categories at baseline.

**Figure 3:**
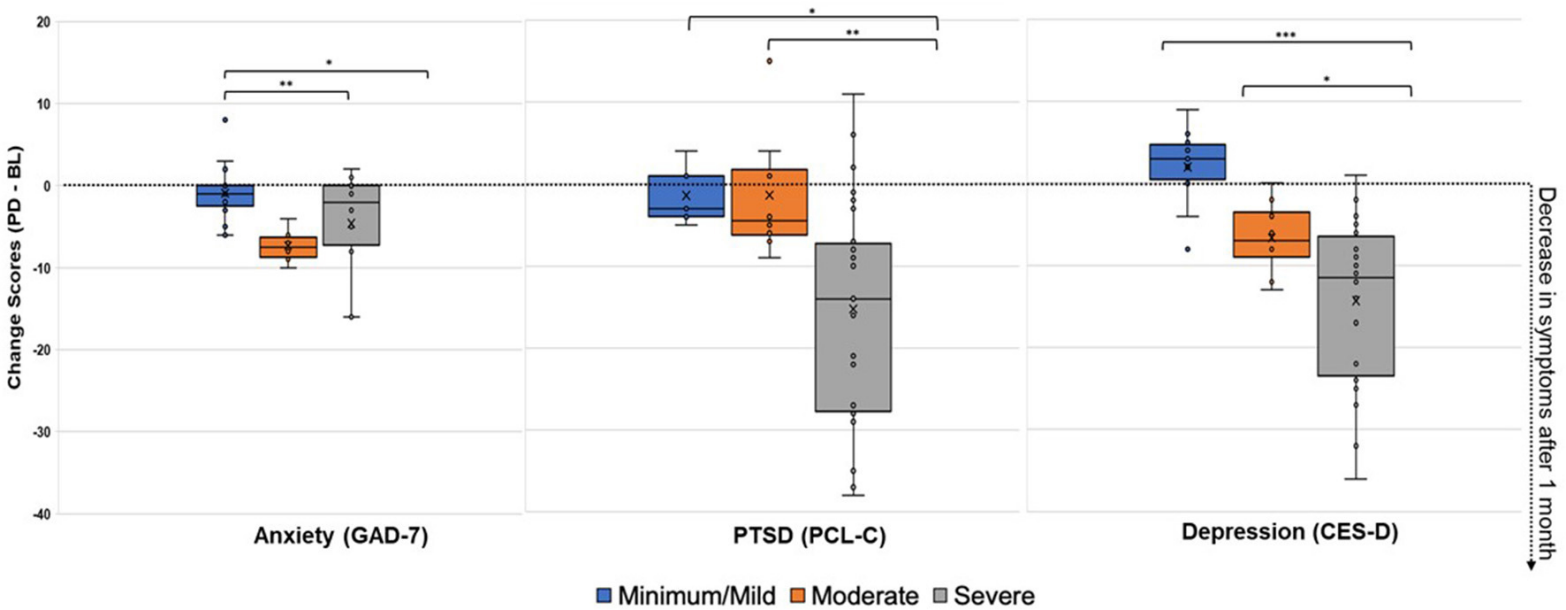
Change Scores After 1-Month on the Low Glutamate Diet (Post-diet minus Pre-diet) for Anxiety, PTSD, and Depression Symptoms. Positive change scores indicate increased reporting of symptoms at post-diet, while negative change scores indicate decreased reporting of symptoms at post-diet (improvement). Minimal/mild, Moderate, and Severe categories were determined by baseline symptom severity. ANOVA LS Mean Difference: ^*^p < 0.05, ^**^p < 0.01, ^***^p < 0.001.

Next, changes across pre-diet baseline symptom severity categories (Figure 2) were assessed. As shown in Table 2, there were significant post-dietary intervention symptom changes between categories captured by the ANOVA for anxiety [*F*_(2, 36)_ = 6.48, *p* = 0.004], PTSD [*F*_(2, 36)_ = 5.89, *p* = 0.006], and depression [*F*_(2, 36)_ = 12.39, *p* < 0.0001]. Specifically, comparisons of LS mean differences were significant for: minimal/mild v. moderate (*t* = 3.27, *p* = 0.002) and minimal/mild v. severe (*t* = 2.27, *p* = 0.029) anxiety groups; minimal/mild v. severe (*t* = 2.39, *p* = 0.022) and moderate v. severe (*t* = 2.89, *p* = 0.007) PTSD groups; and minimal/mild v. severe (*t* = 4.84, *p* < 0.001) and moderate v. severe (*t* = 2.38, *p* = 0.023) depression groups (Figure 3).

**Table 2:** Change in psychological symptoms (Post-diet minus Pre-diet baseline scores) between categories after 1-month on the low glutamate diet (ANOVA).

***N*** **= 39**	**Pre-diet**	**Post-diet**	**Change** **score**	***P*** **-value^a^**
	**Mean (SD)**			
**Anxiety (GAD-7)**				
Minimal/mild (*n* = 23)	4.39 (3.20)	3.48 (3.21)	−0.91 (3.25)	**0.004**
Moderate (*n* = 6)	13.00 (1.26)	5.67 (2.42)	−7.33 (2.16)	
Severe (*n* = 10)	19.00 (1.83)	14.40 (7.70)	−4.60 (6.70)	
**PTSD (PCL-C)**				
Minimal/mild (*n* = 5)	22.40 (3.36)	21.00 (3.74)	−1.40 (3.78)	**0.006**
Moderate (*n* = 8)	33.88 (2.59)	32.50 (7.86)	−1.38 (7.87)	
Severe (*n* = 26)	63.88 (9.28)	48.65 (16.86)	−15.23 (13.54)	
**Depression (CES-D)**				
Minimal/mild (*n* = 9)	11.33 (2.82)	12.89 (5.06)	1.56 (5.08)	**<0.0001**
Moderate (*n* = 7)	18.57 (2.70)	12.86 (3.93)	−5.71 (4.07)	
Severe (*n* = 23)	37.04 (9.12)	22.78 (12.75)	−14.26 (9.97)	

*Change Score, Post-diet minus Pre-diet Baseline Scores*.

a *ANOVA. Bold, significant at the Bonferroni corrected p-value of 0.016*.

### Crossover Challenge With MSG/Placebo

The whole group crossover linear mixed models resulted in no significant main effects for the challenge material (MSG v. placebo), sequence (MSG/placebo v. placebo/MSG), or period (challenge week 1 v. week 2), while the random ID (sequence) term was significant for all models (*p* > 0.001). The insignificant sequence and period effects confirm that there were no carry-over effects, indicating a sufficient washout period between challenge weeks.

The linear mixed model fixed effects (Type-III test) with the addition of the baseline symptom severity categories and interaction with challenge material (MSG v. placebo) can be found in Table 3. For anxiety, there was a significant main effect for symptom severity category (*p* < 0.001) and interaction between challenge material^*^symptom severity category (*p* = 0.016). For PTSD, there was a significant effect for symptom severity category (*p* = 0.009). There were no significant effects for depression.

**Table 3:** Linear mixed model fixed effects (Type-III Test).

**Fixed effect**	***F*** **-value (DF = 35)**	***P*** **-value**
**Anxiety (GAD-7)**		
CM	0.13	0.723
Sequence	0.31	0.582
Period	0.04	0.851
GAD Category	13.59	**<0.001**
CM^*^GAD Category	4.70	**0.016**
**PTSD (PCL-C)**		
CM	0.04	0.840
Sequence	1.29	0.263
Period	1.91	0.176
PCL-C Category	5.46	**0.009**
CM^*^PCL-C Category	0.98	0.387
**Depression (CES-D)**		
CM	0.02	0.887
Sequence	0.01	0.939
Period	0.67	0.420
CES-D Category	2.12	0.136
CM^*^CES-D Category	0.18	0.838

*CM, Challenge Materials (0, placebo, 1, MSG). Sequence: 1, MSG/Placebo, 2, Placebo/MSG. Period: Challenge Week 1 v. Challenge Week 2. Bold, significant at the Bonferroni corrected p-value of 0.016*.

* *indicates interaction*.

**Figure 4:**
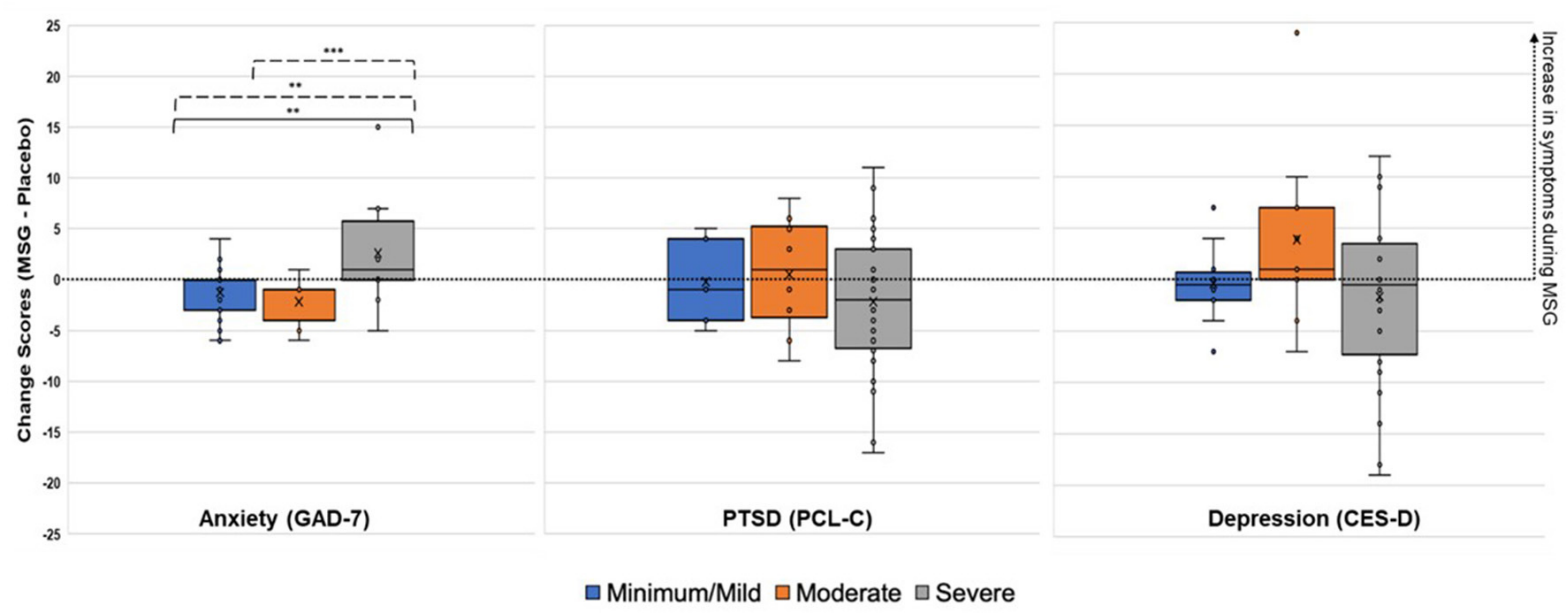
Change Scores during Challenge (MSG minus Placebo) for Anxiety, PTSD, and Depression Symptoms. Positive change score indicates increased reporting of symptoms during MSG challenge, while negative change scores indicate increased reporting of symptoms during placebo. Minimal/mild, Moderate, and Severe categories were determined by baseline symptom severity. ^*^p < 0.05, ^**^p < 0.01, ^***^p < 0.001 (uncorrected). Solid line, LS mean difference during placebo. Dotted line, LS mean difference during MSG.

Due to the significant interaction term for the anxiety model, LS mean differences were compared for challenge material (MSG v. placebo), symptom severity categories, and their interaction (Table 4). At the symptom severity category level, there were significant differences between the minimal/mild v. severe categories (*t* = 5.08, *p* < 0.001) and the moderate v. severe categories (*t* = −3.24, *p* = 0.003). For both, the severe group had higher anxiety symptoms during MSG v. placebo, while the minimal/mild and moderate groups actually showed lower anxiety symptoms during the MSG v. placebo challenge (Figure 4). The LS mean differences across interactions provided further information about the reaction to challenge material within and between severity categories. First, there were no significant differences within minimal/mild or moderate groups when given MSG as compared to placebo, but there was a significant LS mean difference for the severe group, where higher anxiety symptoms were reported during MSG challenge as compared to placebo (*t* = 2.60, *p* = 0.03). Lastly, the comparisons between groups during opposite challenge weeks (e.g., minimal/mild during placebo v. moderate during MSG) further indicated no significant differences between the minimal/mild and moderate groups.

**Table 4:** LS mean difference for treatment (MSG v. Placebo), categories, and their interaction within anxiety (GAD-7).

	**Est**	**SE**	***t-*****value** **(DF=35)**	***p-*** **value**
**CM**				
Placebo v. MSG	−0.25	0.69	−0.36	0.723
**GAD category**				
Min/mild v. moderate	0.67	2.03	0.33	0.743
Min/mild v. severe	7.66	1.51	5.08	**<0.001**
Moderate v. severe	−6.99	2.15	−3.24	**0.003**
CM^*^GAD category				
**Placebo v. MSG: within category**				
Min/mild	−1.30	0.78	−1.66	0.107
Moderate	−2.04	1.62	−1.26	0.217
Severe	2.60	1.15	2.26	**0.030**
**Within placebo**				
Min/mild v. moderate	1.04	2.23	0.47	0.644
Min/mild v. severe	5.71	1.66	3.44	**0.002**
Moderate v. severe	−4.67	2.37	−1.97	0.057
**Within MSG**				
Min/mild v. moderate	0.30	2.23	0.13	0.895
Min/mild v. severe	9.61	1.66	5.79	**<0.001**
Moderate v. severe	−9.31	2.37	−3.92	**<0.001**
**Placebo v. MSG: between categories**				
Min/mild v. moderate	−1.00	2.20	−0.45	0.653
Min/mild v. severe	8.31	1.66	5.00	**<0.001**
Moderate v. severe	7.27	2.37	3.06	**0.004**
**MSG v. placebo: between categories**				
Min/mild v. moderate	−2.34	2.21	−1.06	0.296
Min/mild v. severe	−7.01	1.66	−4.22	**<0.001**
Moderate v. severe	−6.71	2.37	−2.83	**0.008**

*CM, Challenge Materials (0, placebo, 1, MSG). Bold, significant at the p <0.05. Reference category; CM, Placebo (0); GAD Category, Minimal/mild (1)*.

* *indicates interaction*.

## Discussion

To our knowledge, this is the first study to assess the low glutamate diet as a potential adjunct treatment option for psychiatric symptoms among veterans with GWI. The results suggest that the low glutamate diet may be an effective treatment for reducing symptoms of anxiety, PTSD, and depression among veterans with GWI, with significant improvements noted in all three measures after 1 month on the low glutamate diet. Upon challenge with MSG vs. placebo, glutamate (MSG) was only demonstrated to be a direct trigger of anxiety symptoms, and only in those with more severe anxiety. This suggests that other dietary factors, outside of glutamate, such as the increased consumption of antioxidants and micronutrients protective against excitotoxicity, may either alternatively be responsible for the widespread improvement in psychiatric symptoms observed in this study, and/or may be protecting against negative effects from glutamate exposure during the challenge period. More research will be needed to better understand the biological mechanisms being affected. These novel findings support the potential use of the low glutamate diet as an adjunct treatment for psychiatric symptoms among this population.

Psychopharmacological and psychotherapeutic interventions are the typical standard treatments for managing common mental disorders (102–104). Although established efficacy has been noted for these psychiatric treatments, many have not achieved remission or found adequate relief of symptoms after use (105, 106). Additionally, access to these treatments and negative side effects can affect adherence to these strategies long-term (107). Therefore, finding alterative or adjunct treatments to address these symptoms is of the utmost importance for GW veterans and the population at large.

Unlike medication effects, which are usually aimed at altering action of one receptor or enzyme to ameliorate psychiatric symptoms [e.g., serotonergic pathway, glutamatergic pathway (57, 108)], the low glutamate diet aims to reduce glutamatergic excitotoxicity while concurrently increasing consumption of nutrients known to be protective against excitotoxicity, with the aim of improving neurotransmission (17, 28, 69, 109).

In this study, variability in the response to the acute challenge with MSG vs. placebo may have been influenced by underlying nutritional intake that could be altering how glutamate is handled in the CNS. For example, glutamate is the precursor compound for the production of the inhibitory neurotransmitter GABA (110). Glutamate decarboxylase is the enzyme which converts glutamate to GABA, and it necessitates vitamin B6 [in the form of pyridoxal phosphate (PLP)] as a cofactor for it to function (94, 110). Riboflavin is needed for conversion of dietary vitamin B6 into its active PLP form, and also has its own neuroprotective properties against excitotoxicity, as well as antioxidant functions (92). Glutamate is also a substrate (along with cysteine and glycine) in the production of the endogenous antioxidant glutathione (111, 112). Thus, optimal production of glutathione can both reduce glutamate levels and increase antioxidant function. The combination of lowering glutamate levels, while also providing adequate or increased micronutrient intake, may alter how the body handles the glutamate challenge, while simultaneously increasing antioxidant function, thereby ameliorating the downstream effects on oxidative stress (100, 101). Finally, reduced excitotoxicity and improved overall handling of glutamate could be achieved through improved metabolism (e.g., increased ATP production) in astrocytes, which are responsible for the uptake of glutamate from the synaptic cleft (113). Future research will be needed to explore each of these potential mechanisms individually or in tandem with each other as protection against glutamate exposure.

Emerging research has demonstrated the importance of nutrient intake and dietary composition of whole foods for optimizing mental health (114–117). The results of this study are in line with multiple meta-analyses, systematic reviews, and observational studies demonstrating positive effects of healthy dietary intake on mental health outcomes (118–121). Additionally, a few RCT studies have also reported significant improvement in depression and anxiety symptoms from Mediterranean-style dietary interventions (122–124). In the SMILES trial, only participants with major depressive episodes were included, and they found significant improvement in depression and noted 32.3% of their sample achieved remission after 12 weeks on the diet (122). This is consistent with our study findings where 23% of our sample no longer met the threshold for depression after 1 month on the low glutamate diet. Another group conducted an RCT and analyzed data from the intervention group consuming a Mediterranean-style diet and identified reduced consumption of processed food as the most impactful component for the improvements in depression observed among this group (124). Additionally, a scoping review found that unhealthy dietary patterns and high artificial sweetener intake (which sometimes contain excitotoxins) were associated with increased anxiety outcomes (118). To the authors' knowledge, no RCTs have been conducted focusing solely on diet and PTSD. However, cross-sectional studies, often not looking at dietary intake as the main outcome, have reported that individuals with PTSD had lower diet quality and higher intake of sodas and fast-food (125, 126). Thus, these studies highlight similarities in psychiatric symptom reduction during dietary interventions that improve nutrient intake and diet quality, while removing processed foods from the diet.

Strengths of this research include a diverse geographic recruitment of participants and a sample which includes members from multiple military branches and a large proportion of female participants, which is uncommon in veteran research. There were more women in the GW than in any other prior conflict (127), thus inclusion of women in GWI research is essential. Additionally, the study design allowed assessment of overall dietary effects, as well as the acute effects of challenge with MSG in a double-blind, placebo-controlled crossover fashion. However, this study is limited by its small sample size and these results are not generalizable outside of veterans with GWI. Additionally, psychiatric symptoms were assessed solely by questionnaire rather than clinical interview in this pilot study; thus, future research may benefit from the inclusion of a clinical evaluation as well (76–81, 83). Future research is also needed to: confirm these findings in a larger group of veterans with GWI, test the longer-term effects of the dietary intervention, explore the mechanisms behind the observed effects, and to test the effectiveness of the low glutamate diet as a treatment for anxiety, PTSD, and depression in the general population.

In conclusion, anxiety, PTSD, and depression are common comorbidities in veterans who suffer from GWI, and these psychiatric symptoms may improve by following the low glutamate diet. There is biological plausibility for how the low glutamate diet may be able to affect not only excitotoxicity, but also corresponding oxidative stress and inflammation that may be potentiating these symptoms. If these results hold in a larger clinical trial, the low-glutamate diet may be an effective low-cost adjunct treatment option for treating psychiatric symptoms in veterans with GWI.

## Data Availability Statement

The raw data supporting the conclusions of this article will be made available by the authors, without undue reservation.

## Ethics Statement

The studies involving human participants were reviewed and approved by the Institutional Review Boards at American University and Georgetown University. The patients/participants provided their written informed consent to participate in this study.

## Author Contributions

KH and JB: conceptualization, design of the work, and resources. KH: investigation and supervision. EB and AK: acquisition of data and data curation. KH and MB: methodology. AK, MB, and KH: analysis. EB, AK, and KH: visualization and writing—original draft preparation. EB, AK, MB, JB, and KH: writing—review and editing. KH and EB: project administration. KH, MB, and JB: funding acquisition. All authors have read and approved the version to be published and agree to be accountable for all aspects of the work.

## Funding

The U.S. Army Medical Research Acquisition Activity, 820 Chandler Street, Fort Detrick MD 21702-5014 is the awarding and administering acquisition office. This work was supported by the Office of the Assistant Secretary of Defense for Health Affairs, through the Gulf War Illness Research Program under Award No. W81XWH-17-1-0457.

## Author Disclaimer

Opinions, interpretations, conclusions and recommendations are those of the authors and are not necessarily endorsed by the Department of Defense.

## Conflict of Interest

The authors declare that the research was conducted in the absence of any commercial or financial relationships that could be construed as a potential conflict of interest.

## Publisher's Note

All claims expressed in this article are solely those of the authors and do not necessarily represent those of their affiliated organizations, or those of the publisher, the editors and the reviewers. Any product that may be evaluated in this article, or claim that may be made by its manufacturer, is not guaranteed or endorsed by the publisher.
