# Supplementary material for: The Effect of the Low Glutamate Diet on the Reduction of Psychiatric Symptoms in Veterans With Gulf War Illness: A Pilot Randomized-Controlled Trial
Source: Front Psychiatry. 2022 Jun 20;13:926688. doi: 10.3389/fpsyt.2022.926688 (PMC9251130; doi:10.3389/fpsyt.2022.926688)
Supplement: Supplementary file 1 [file Data_Sheet_1.DOC]

**
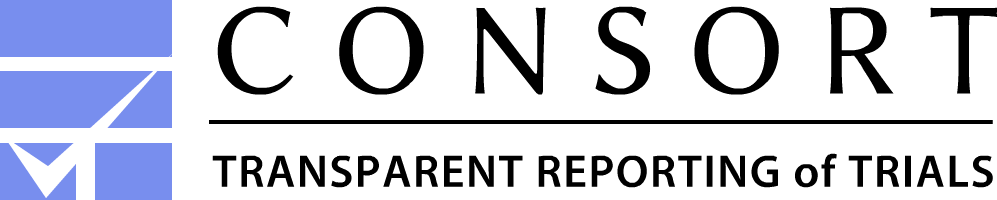
**

**CONSORT 2010 Flow Diagram**

**Allocation**

**Analysis**

**Follow-Up**

**Enrollment**

Assessed for eligibility (n=212)

Excluded (n=100)

♦  Not meeting inclusion criteria (n=49)

♦  Declined to participate (n=51)

♦  No time now, but interested in a future study (N=112)

Analysed (n=20)

Lost to follow-up (n=0)

Allocated to intervention (n=23)

♦ Received allocated intervention (n=20)

♦ Disqualified after arrival (n=1)

♦ Dropped out prior to intervention

- Lack of computer/resources (n=1)
- Lack of self-efficacy (n=1)

Lost to follow-up (n=0)

Allocated to intervention (n=23)

♦ Received allocated intervention (n=20)

♦ Dropped out due to:

- Kidney failure (n=1)
- Gastric bypass (n=1)
- Major car accident (n=1)

Analysed (n=20)

Randomized (n=46)
